# Cadmium tolerance mediated by *GhBCAT12* through branched-chain amino acid degradation via vacuolar sequestration in cotton

**DOI:** 10.1007/s44154-026-00335-z

**Published:** 2026-08-10

**Authors:** Lei Xiao, Xiugui Chen, Yunxin He, Depei Kong, Junjuan Wang, Jihua Yang, Yupeng Cui, Hui Huang, Kang Zhao, Jing Wang, Hao Lan, Ruize Song, Fange Wu, Xinrui Zhang, Xin Yu, Junshan Zhu, Jinkai Liu, Songyue Zhou, Xiaoyu Tian, Mengru Wei, Shuai Wang, Lixue Guo, Ping Li, Xiaowei Wang, Xue-Rong Zhou, Wuwei Ye

**Affiliations:** 1 Institute of Cotton Research of Chinese Academy of Agricultural Sciences / Anyang Research Base, State Key Laboratory of Cotton Bio-Breeding and Integrated Utilization, Anyang Institute of Technology / National Center of Technology Innovation for Comprehensive Utilization of Saline-Alkali Land, Anyang, Henan 455000 China; 2 Yuelushan Laboratory/Institute of Cotton and Sericulture, Hunan Academy of Agricultural Sciences, Changsha, Hunan 410128 China; 3 CSIRO Agriculture and Food, PO Box 1700, Canberra, ACT 2601 Australia; 4 Shandong Zhongli Cotton Industry Technology Co., Ltd, Dongying, Jinan 257500 China; 5 Institute of Agricultural Science of 13th Division of Xinjiang Production and Construction Corps, Hami, Xinjiang 839000 China

**Keywords:** Branched-chain amino acids, Cd^2+^ stress, Acetyl-CoA, ATP, *Gossypium hirsutum*

## Abstract

Soil Cd^2+^ pollution threatens agricultural safety. Branched-chain amino acid (BCAA) metabolism is involved in stress responses, but its role in cotton Cd^2+^ tolerance remains unclear. Here, 16 *GhBCAT* genes were identified in *Gossypium hirsutum*, among which *GhBCAT12* was significantly induced by Cd^2+^ stress. Silencing *GhBCAT12* increased Cd^2^^+^ sensitivity, causing growth inhibition, oxidative damage, and cell death. Metabolomic analysis revealed the accumulation of leucine, isoleucine, and valine, with decreased acetyl-CoA, indicating that *GhBCAT12* mediates BCAA degradation. This study reveals a novel pathway in which *GhBCAT12*-mediated BCAA degradation provides acetyl-CoA precursors for the tricarboxylic acid (TCA) cycle, sustaining ATP production, which in turn powers vacuolar Cd^2^^+^ sequestration. These findings provide a new target for improving heavy metal tolerance in crops.

**Supplementary Information:**

The online version contains supplementary material available at 10.1007/s44154-026-00335-z.

## Introduction

Cd^2+^ is a highly toxic heavy metal. In recent years, driven by rapid industrialization and excessive agrochemical use, heavy metal contamination of soils has become increasingly severe in China. Due to its high water solubility and soil-to-plant transfer coefficient, Cd^2^^+^ is readily absorbed by crop roots and accumulated (Li et al. 2026). Cd^2^^+^ disrupts physiological processes such as photosynthesis and mineral nutrient uptake, significantly inhibiting the normal growth and development of crops, leading to yield reduction (Hu et al. 2022; Liu et al. 2023; Zhu et al. 2026). More importantly, Cd^2^^+^ can accumulate in edible plant parts and transfer along the food chain, thereby posing a serious threat to agricultural product safety, food security, and ultimately public health (Yu et al. 2024b).

Amino acids are not only the fundamental building blocks of proteins but also play multiple key roles in plant stress resistance, acting directly or indirectly as signaling molecules, osmotic regulators, precursors of antioxidants, and substrates for the synthesis of secondary metabolites. Under abiotic stress, the content and composition of amino acids in plants undergo significant changes (Fu et al. 2018). Studies have shown that amino acids play an important role in plant stress tolerance (Ingrisano et al. 2023). For instance, in research on Cd^2^^+^ tolerance in cotton, cysteine alleviates Cd^2^^+^ toxicity by increasing glutathione levels and reducing oxidative damage (Meng et al. 2023). In rice, glutamate inhibits Cd^2^^+^ uptake and translocation by suppressing the expression of Cd^2^^+^ transporter genes in roots, thereby mitigating Cd^2^^+^ toxicity (Jiang et al. 2020). Proline, in addition to functioning as an osmotic regulator, is also a potent scavenger of reactive oxygen species (ROS); under Cd^2^^+^ stress in soybean, it enhances Cd^2^^+^ tolerance by reducing ROS levels (Wang et al. 2025). BCAAs, including valine, leucine and isoleucine, exhibit varying degrees of accumulation under diverse abiotic stress conditions (Cosio and Renault 2020; Zemanová et al. 2017). BCAAs not only function as osmolytes in plant abiotic stress responses (Shim et al. 2023) but also serve as precursors for the TCA cycle intermediates succinyl-CoA and acetyl-CoA, as well as direct electron donors for the mitochondrial electron transport chain (Araújo et al. 2010; Kirma et al. 2012). For plants experiencing carbon starvation, the catabolism of free BCAAs is a core physiological process essential for maintaining energy homeostasis and survival under stress (Izumi and Ishida 2019). BCAT (branched-chain amino acid aminotransferase) plays a critical role in BCAA metabolism, as it catalyzes both the final step of BCAA biosynthesis and the initial step of BCAA degradation (Angelovici et al. 2013). *BCATs* have been shown to play important roles in plant stress responses across various species, such as rice and barley. For example, in rice, *OsBCAT2*, a gene responsible for BCAA degradation, positively regulates salt tolerance (Sun et al. 2024). *OsDIAT* positively regulates drought tolerance in rice by promoting BCAA biosynthesis (Shim et al. 2023). In barley, *HvBCAT-1*, which is potentially involved in BCAA degradation, is upregulated under drought stress (Malatrasi et al. 2006).

Cotton, a major cash crop and raw material for the textile industry, exhibits a strong capacity for the absorption, translocation, and accumulation of Cd^2+^ (Yu et al. 2024a). Compared to staple food crops such as wheat and rice, cotton significantly reduces the risk of Cd^2+^ entering the food chain (Wei et al. 2024; Zhang et al. 2025b), making it a suitable candidate for cultivation in Cd^2+^-contaminated agricultural soils.

Previous data from our laboratory revealed that the degradation pathways of valine, leucine, and isoleucine were significantly enriched under Cd^2^^+^ stress, and that *GhBCATs* were positively responsive to Cd^2^^+^ stress (Han et al. 2019). This trend is consistent with previous findings (Li et al. 2022). However, whether *GhBCATs* participate in cotton Cd^2^^+^ tolerance remains unclear. We hypothesized that *GhBCAT*-mediated BCAA metabolism contributes to cotton adaptation to Cd^2^^+^ stress. In this study, members of the *BCAT* gene family in *Gossypium hirsutum* were identified, and their expression profiles under Cd^2^^+^ stress were characterized, and the function of *GhBCAT12* was verified. Our results reveal a critical mechanism linking BCAA catabolism, energy homeostasis, and vacuolar Cd^2^^+^ sequestration, providing a novel target for enhancing heavy metal tolerance in crops.

## Results

### Identification and characterization of the *GhBCAT* gene family

To investigate the potential roles of BCAA metabolism in cotton Cd^2^^+^ tolerance, *BCAT* family members were first identified in four cotton species. A total of 47 *BCAT* genes were identified, with the two tetraploid species (*Gossypium hirsutum* and *Gossypium barbadense*) containing approximately twice as many members (16 and 15, respectively) as the diploid progenitors (*Gossypium arboreum* and *Gossypium raimondii*, each with 8 members), suggesting that whole-genome duplication events contributed to the expansion of this family (Table S1). The physicochemical properties of the 16 GhBCAT proteins varied considerably, with amino acid lengths ranging from 242 to 560, molecular weights ranging from 26.8 to 63.1 kDa, and isoelectric points ranging from 5.6 to 8.7 (Table S2).

**Fig. 1:**
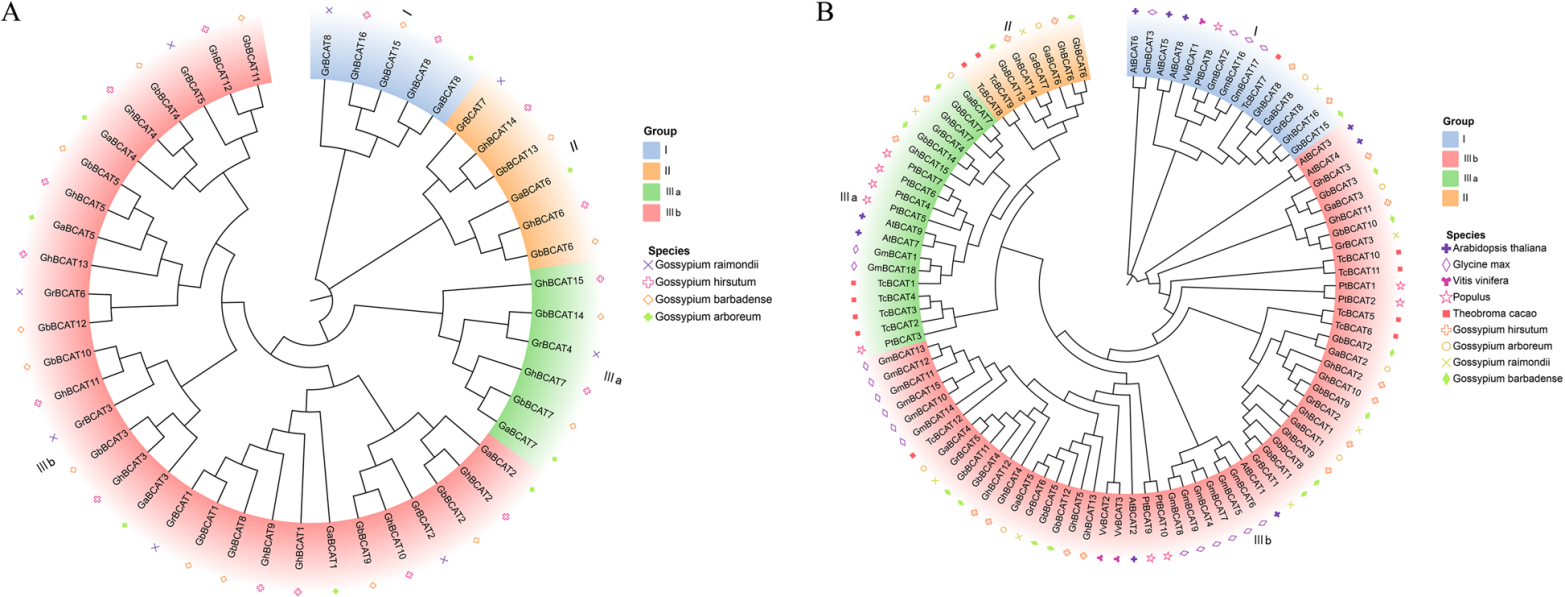
Phylogenetic relationships of the *BCAT* family in nine plant species. **A** Phylogenetic relationships of the *BCAT* family in four cotton species (*Gossypium hirsutum*, *Gossypium barbadense*, *Gossypium arboreum*, and *Gossypium raimondii*). **B** Phylogenetic relationships of the *BCAT* family in the four cotton species and five other plant species (*Arabidopsis thaliana*, *Glycine max*, *Vitis vinifera*, *Populus trichocarpa*, and *Theobroma cacao*)

To further characterize this gene family, phylogenetic analysis of BCAT proteins from cotton and other plant species (including *Arabidopsis thaliana*, *Glycine max*, *Populus trichocarpa*, *Vitis vinifera*, and *Theobroma cacao*) was performed, and the family was found to be divided into three distinct clades (Groups I-III) (Fig. 1). Chromosomal localization analysis showed that *GhBCAT* genes are distributed across 12 chromosomes in *Gossypium hirsutum*, with homologous copies present on both the At and Dt subgenomes, further supporting an allopolyploidization-driven expansion (Fig. S1). Conserved motif and gene structure analyses revealed that Group I members lack several characteristic motifs and exhibit simpler structures compared with Groups II and III, which retain nine to ten conserved motifs. Notably, three motifs (Motifs 2, 5, and 8) were universally present across all *GhBCAT* members, establishing them as signature core motifs of this family (Fig. S2).

Finally, to explore the potential regulatory mechanisms of these genes, promoter sequence analysis was performed using the PlantCARE database. The results revealed the presence of multiple hormone-responsive elements (related to MeJA, auxin, gibberellin, abscisic acid, and salicylic acid) as well as stress-responsive elements (associated with hypoxia, drought, low temperature, and general stress responses) (Fig. S3). These results provide a key bioinformatics foundation for further dissecting the regulatory functions of *GhBCAT* genes within hormone-stress interaction networks.

### *GhBCAT*s gene expression and qRT-PCR analysis

**Fig. 2:**
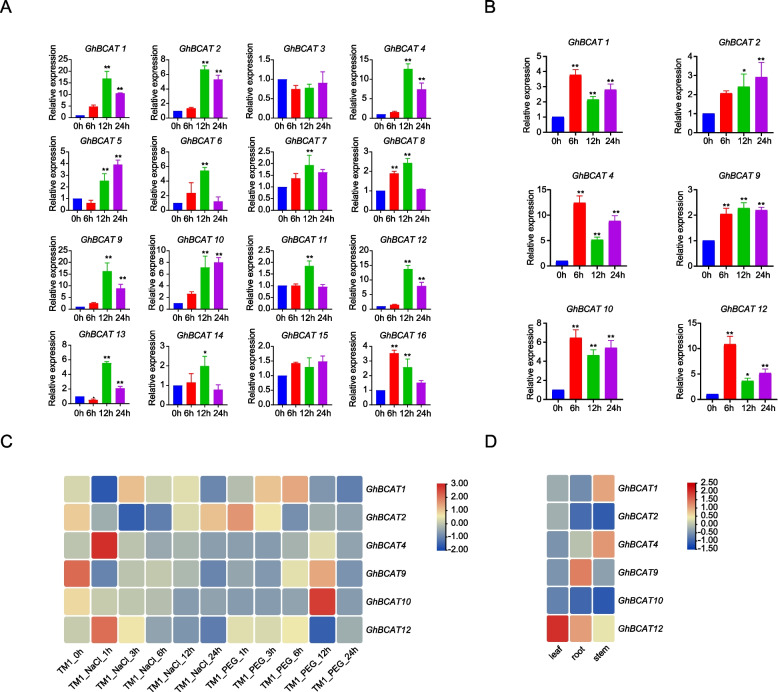
Gene expression profile analysis. **A** Expression profiles of *GhBCAT*s in leaves under 4 mM Cd^2^^+^ stress. **B** Expression profiles of *GhBCAT*1/2/4/9/10/12 in roots under 4 mM Cd^2^^+^ stress. **C** Expression profiles of *GhBCAT*s under abiotic stresses (salt, PEG). Data were obtained from Cotton Omics Database. **D** Expression profiles of *GhBCAT*s in different tissues (leaf, root, stem) under non-stress conditions. Data were obtained from Cotton Omics Database. For panels **A** and **B**, Data are presented as mean ± SD (*n* = 3). Statistical significance was determined by one‑way ANOVA followed by Tukey's HSD test; different letters indicate significant differences at *p* < 0.05

To investigate whether *GhBCAT*s are involved in regulating the response of cotton to Cd^2+^ stress, this study examined the transcriptional dynamics of *GhBCAT* family genes in cotton leaves under Cd^2+^ stress using qRT-PCR. The results showed that most *GhBCAT* genes exhibited significant responses to Cd^2+^ stress, among which the expression levels of *GhBCAT1, GhBCAT2*, *GhBCAT4*, *GhBCAT9*, *GhBCAT10*, and *GhBCAT12* were markedly higher than those of other members, all showing significant upregulation after 12 h of Cd^2^^+^ stress (Fig. 2A). Further expression analysis in root tissue revealed that *GhBCAT4* and *GhBCAT12* were particularly prominent (Fig. 2B). Considering the expression patterns of family members under various stress conditions and their tissue expression profiles, *GhBCAT12* demonstrated stronger stress responsiveness compared with other members and maintained relatively high expression levels in roots, stems, and leaves (Fig. 2C-D). Therefore, *GhBCAT12* was selected as a candidate gene for subsequent functional validation.

### Subcellular localization

**Fig. 3:**
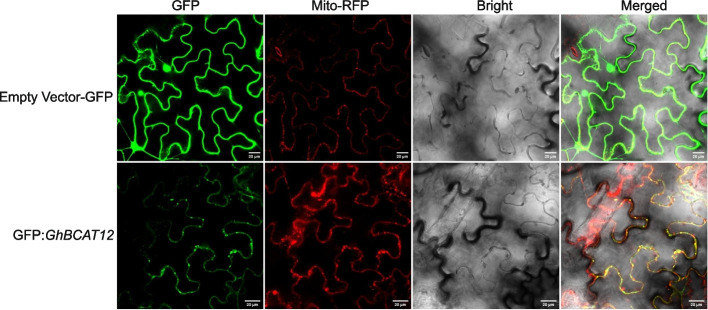
Subcellular localization of the GhBCAT12 protein

The function of BCATs depends on their subcellular localization in plants. To determine the subcellular localization of GhBCAT12, prediction was initially performed via Target P-2.0. The non-plant model indicated that the protein contains a high-confidence mitochondrial transit peptide (mTP = 0.5244). Although the plant model yielded relatively complex results due to the need to distinguish chloroplast signals, the scores for secretion pathway and thylakoid lumen localization were extremely low (SP = 0.0102; thylakoid = 0.0027), suggesting that the mitochondrion is its potential localization site. To validate this prediction, a *GhBCAT12*-GFP fusion expression vector was constructed, and mitochondrial co-localization experiments were performed. The results showed that the green fluorescence of *GhBCAT12*-GFP completely overlapped with the red fluorescence of MitoTracker Red (Fig. 3), confirming that the GhBCAT12 protein is localized in the mitochondrion.

### *GhBCAT12* positively regulates cotton tolerance to Cd^2^⁺ stress

**Fig. 4:**
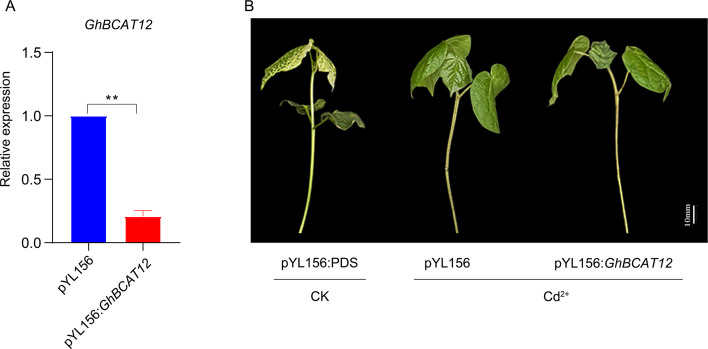
Effects of silencing *GhBCAT12* on cotton seedlings under Cd^2^^+^ stress. **A** Determination of gene silencing efficiency. Data are presented as mean ± SD (*n* = 3). Statistical significance was determined by unpaired two‑tailed Student's t‑test (**p < 0.01). **B** Growth phenotypes of pYL156:PDS positive control and pYL156 control versus pYL156:*GhBCAT12*-silenced plants under Cd^2^^+^ stress

The *GhBCAT12* gene was silenced using VIGS (virus-induced gene silencing) technology. Successful establishment of the silencing system was confirmed when cotton plants infiltrated with pYL156:PDS exhibited an albino phenotype (Fig. 4B). At this stage, transcript levels of *GhBCAT12* in *Gossypium hirsutum* leaves were quantified by qRT-PCR. The results showed that gene expression levels in the pYL156:*GhBCAT12* treatment group were significantly reduced compared with the pYL156 empty vector group, indicating that *GhBCAT12* was effectively silenced (Fig. 4A).

Phenotypic analysis was conducted on the pYL156 empty vector control group and pYL156:*GhBCAT12*-silenced lines treated with 4 mM Cd^2^^+^. Under the same stress duration, the pYL156:*GhBCAT12*-silenced plants displayed phenotypes such as leaf dehydration and wilting earlier than the control group (Fig. 4B).

To further elucidate the molecular mechanism by which *GhBCAT12* mediates the Cd^2^^+^ stress response in cotton, physiological changes were systematically compared between silenced lines and control plants under Cd^2^^+^ stress.

**Fig. 5:**
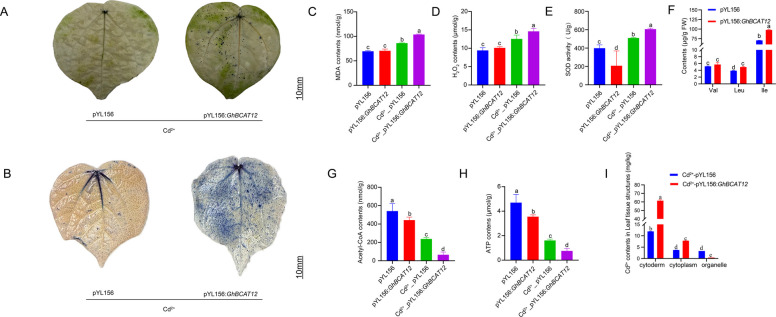
Effects of silencing *GhBCAT12* on cotton under Cd^2^^+^ stress. **A** NBT staining. **B** Trypan blue staining. **C-E** MDA, H₂O₂, SOD contents in cotton plants. **F** Contents of Valine, Leucine, and Isoleucine in pYL156 control plants and pYL156:*GhBCAT12* plants under non-stress conditions. **G-H** Acetyl-CoA, ATP contents. **I** Cd^2+^ contents in leaf subcellular fraction of *GhBCAT12*-silenced and pYL156 control plants under Cd^2+^ stress. Data are presented as mean ± SD (*n* = 3 biological replicates, with each replicate consisting of 5 plants). Statistical significance was determined by one‑way ANOVA followed by Tukey's HSD test; different letters indicate significant differences at *p* < 0.05

In terms of oxidative stress response, the contents of MDA and H₂O₂ were significantly accumulated in the silenced plants after Cd^2+^ treatment (Fig. 5C-D). Further validation through histochemical staining revealed that NBT staining showed a denser distribution of blue formazan precipitate in the leaves of silenced plants, indicating increased generation of superoxide anion (O₂⁻). Trypan blue staining revealed a significantly enlarged stained area in their leaves, confirming elevated cell mortality and aggravated membrane damage (Fig. 5A-B). These findings were consistent with the observed phenotypic changes in the plants. Notably, SOD activity was significantly increased in the silenced plants (Fig. 5E), suggesting that plants partially alleviate Cd^2^^+^-induced oxidative damage by activating the antioxidant enzyme system.

Analysis of the amino acid metabolic profile revealed that the BCAA metabolism was specifically altered in the silenced plants. The contents of leucine, isoleucine, and valine were all increased, with leucine and isoleucine showing significant increases (Fig. 5F). These results suggest that *GhBCAT12* may be involved in the degradation of BCAAs, particularly leucine and isoleucine. However, it should be noted that this conclusion is derived from indirect in vivo evidence, and the substrate preference awaits further validation through in vitro enzyme activity assays.

As BCAA degradation contributes acetyl-CoA precursors to the TCA cycle, leaf acetyl-CoA contents were further determined. The results showed that under Cd^2^^+^ stress, acetyl-CoA levels in pYL156:*GhBCAT12* plants were significantly lower than those in pYL156 plants (Fig. 5G). Acetyl-CoA acts as a substrate for the TCA cycle, and the adequacy of its supply directly affects mitochondrial ATP production efficiency. Accordingly, ATP contents in leaves were determined under different treatments. The results showed that the trend of ATP contents was consistent with that of acetyl-CoA (Fig. 5H).

To investigate the link between BCAA catabolism and mitochondrial energy production, transcriptional changes in key TCA cycle enzymes were examined. Under Cd^2^^+^ stress, transcript levels of two key TCA cycle enzymes, citrate synthase (*GhCS6*) and isocitrate dehydrogenase (*GhIDH1*) (Table S3), showed no significant changes between the silenced plants and the pYL156 control (Fig. S4A-B), ruling out transcriptional suppression as the primary cause. However, the NADH/NAD⁺ ratio was significantly lower in the silenced plants than in the control (Fig. S4C). Given the reduced acetyl-CoA levels observed in these plants (Fig. 5G), this decrease in the NADH/NAD⁺ ratio is most consistent with constrained TCA cycle flux resulting from limited carbon substrate supply. Collectively, these data are most consistent with the interpretation that the decreased ATP contents in *GhBCAT12*-silenced plants may arise from insufficient acetyl-CoA supply due to impaired BCAA degradation, which in turn limits TCA cycle flux and mitochondrial ATP synthesis.

The normal function of vacuolar cadmium transporters is strictly dependent on ATP supply. Therefore, a decrease in ATP levels may directly lead to a reduction in vacuolar sequestration capacity (He et al. 2019). To further validate the impact of *GhBCAT12*-mediated energy metabolism on Cd^2+^ compartmentalization, Cd^2^^+^ contents in different subcellular fractions were determined. The results showed that under 4 mM Cd^2^^+^ stress, compared with the empty vector control, *GhBCAT12*-silenced plants exhibited significantly higher Cd^2+^ content in the cell wall and soluble fractions, while Cd^2^^+^ accumulation in the organelle fraction was significantly reduced (Fig. 5I).

**Fig. 6:**
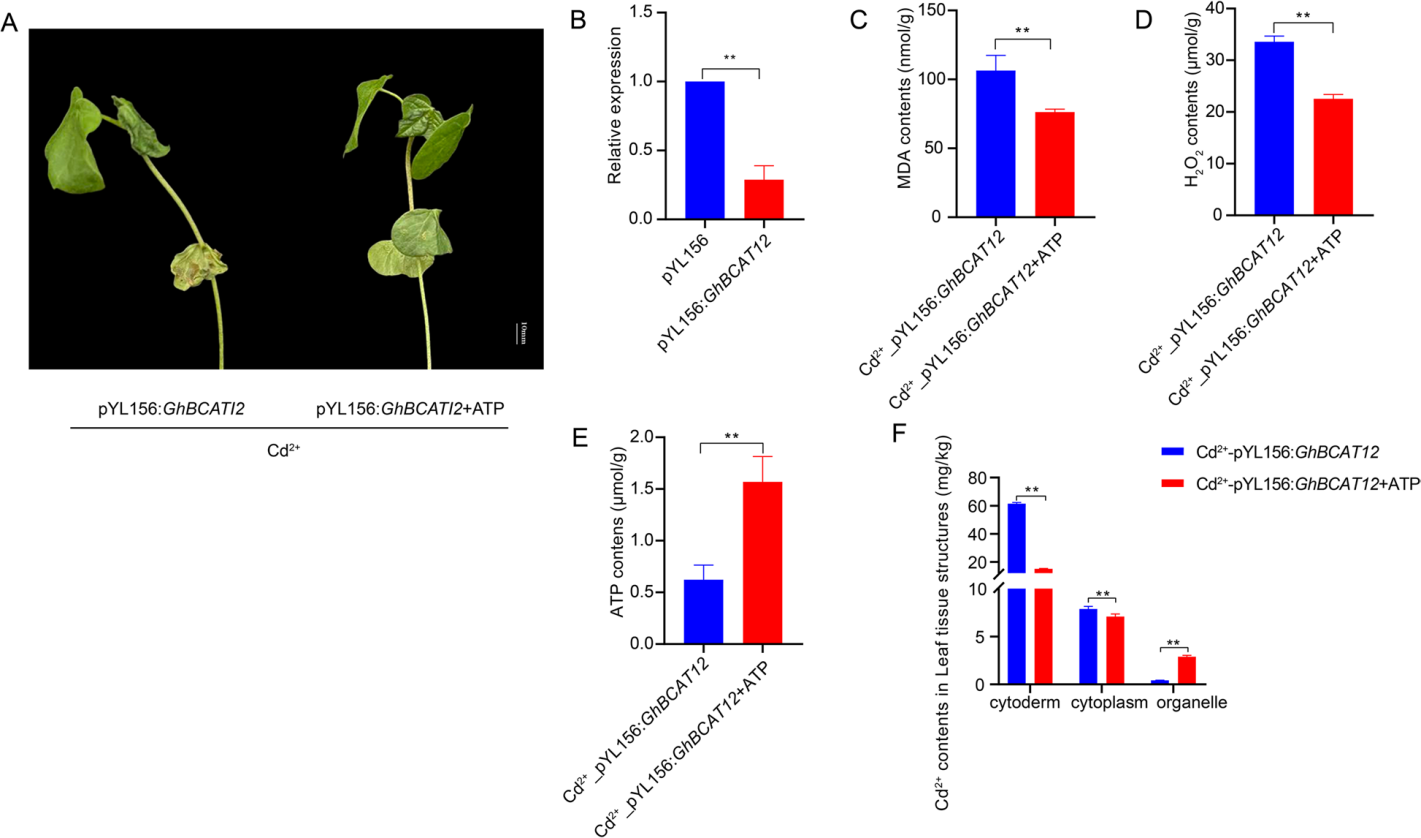
Effects of exogenous ATP supplementation on Cd^2^^+^ tolerance in *GhBCAT12*-silenced cotton plants. **A** Phenotypic analysis of pYL156:*GhBCAT12* plants treated with 4 mM Cd^2^^+^ and 4 mM Cd^2^^+^ combined with 400 µM ATP. **B** Determination of gene silencing efficiency. **C-E** MDA, H₂O₂ and ATP contents. **F** Subcellular Cd^2^^+^ distribution. Data are presented as mean ± SD (*n* = 3 biological replicates, with each replicate consisting of 5 plants). Statistical significance was determined by Student's t‑test (**p < 0.01)

To further verify the causal relationship between ATP depletion and reduced vacuolar Cd^2^^+^ sequestration capacity, exogenous ATP supplementation experiments were performed. The optimal concentration of exogenous ATP was first determined through a dose‑response assay. Among the tested concentrations, 400 µM ATP was found to most effectively alleviate the Cd^2^^+^‑induced phenotypic defects in *GhBCAT12*‑silenced plants, including leaf dehydration and wilting (Fig. S5). Consistent with the phenotypic observations, exogenous ATP supplementation significantly reduced the accumulation of MDA and H₂O₂ in the silenced plants (Fig. 6C-D), indicating effective mitigation of oxidative damage. Furthermore, ATP contents were significantly restored in the silenced plants under Cd^2^^+^ stress following exogenous ATP supplementation (Fig. 6E). Subcellular Cd^2^^+^ distribution analysis further revealed that exogenous ATP treatment enhanced Cd^2^^+^ sequestration in the vacuolar (organellar) fraction, while reducing Cd^2^^+^ accumulation in the cytoplasmic fraction (Fig. 6F). Collectively, these results support the conclusion that exogenous ATP supplementation effectively rescues the Cd^2^^+^‑sensitive phenotype of *GhBCAT12*‑silenced plants, alleviates oxidative damage, and restores cellular energy levels.

## Discussion

### *GhBCAT12* is a key metabolic regulator in the response of cotton to Cd^2^⁺ stress

The role of BCAA metabolism in plant stress responses has garnered increasing attention (Qiang et al. 2025; Zhang et al. 2025a); however, its function in heavy metal stress remains to be fully elucidated. This study found that under Cd^2^^+^ stress, the expression of multiple *GhBCAT* genes in cotton leaves underwent significant changes, among which *GhBCAT12* maintained relatively high expression levels in roots, stems, and leaves and showed the most pronounced response to Cd^2^^+^ stress. Silencing *GhBCAT12* using VIGS technology significantly enhanced the Cd^2^^+^ sensitivity of cotton, manifested by more severe growth inhibition, oxidative damage, and cell death, confirming that *GhBCAT12* is a positive regulator of Cd^2^^+^ tolerance in cotton. This finding echoes studies showing that *OsBCAT2* positively regulates salt tolerance (Sun et al. 2024) and *OsDIAT* positively regulates drought tolerance in rice (Shim et al. 2023), suggesting that *BCAT* family genes may play conserved and critical regulatory roles in plant responses to diverse abiotic stresses.

Genome-wide analysis identified 16 *GhBCATs* in *Gossypium hirsutum*, and phylogenetic analysis placed *GhBCAT12* and *GhBCAT4* into distinct subclades (Fig. 1), suggesting potential divergence in their regulatory or catalytic properties. Interestingly, despite this phylogenetic distance, *GhBCAT4* exhibited expression patterns under Cd^2^^+^ stress that were highly similar to those of *GhBCAT12*, including significant upregulation and high transcript abundance in both leaves and roots (Fig. 2A-B). This observation raises the possibility that *GhBCAT4* might act redundantly or synergistically with *GhBCAT12* in response to Cd^2^^+^ stress. In addition, other Cd^2^^+^-responsive members, such as *GhBCAT1*, *GhBCAT2*, *GhBCAT9* and *GhBCAT10* (Fig. 2A), displayed differential expression patterns across roots, stems and leaves (Fig. 2D), suggesting that they may participate in Cd^2^^+^ tolerance in a tissue‑specific manner.

### Mitochondrial localization of GhBCAT12 aligns with its function

The function of BCATs depends on their subcellular localization in plants (Angelovici et al. 2013). In this study, through a combination of Target P-2.0 prediction and co-localization experiments, GhBCAT12 protein was confirmed to be localized in the mitochondria (Fig. 3). In plants, the subcellular localization of BCAT enzymes is closely associated with their metabolic direction: BCATs localized in chloroplasts are primarily involved in the biosynthesis of BCAAs (Angelovici et al. 2013; Diebold et al. 2002), whereas those localized in mitochondria are mainly responsible for their degradation (Angelovici et al. 2013). This finding is highly consistent with the accumulation of leucine and isoleucine observed upon *GhBCAT12* silencing, further supporting the role of *GhBCAT12* in BCAA degradation within the mitochondria. As the primary site of BCAA oxidation, the mitochondrion enables GhBCAT12 to directly supply acetyl-CoA precursors to the TCA cycle, providing a cellular basis for the subsequent reprogramming of energy metabolism.

### *GhBCAT12*-mediated metabolic reprogramming of energy metabolism is key to Cd^2^⁺ tolerance

Considering the mitochondrial localization of GhBCAT12 and its involvement in BCAA catabolism, the impact of its silencing on energy metabolism was further explored. Under Cd^2^^+^ stress conditions, plant energy metabolic homeostasis is significantly disrupted (Wang et al. 2021). The burst of ROS triggered by Cd^2^^+^ exposure (including hydrogen peroxide, superoxide anion, and hydroxyl radicals) is a core factor contributing to Cd^2^^+^ toxicity in plants (El Rasafi et al. 2022; Rizwan et al. 2016). Oxidative stress induces a reprogramming of cellular metabolic flux, shunting glycolytic flux toward the oxidative pentose phosphate pathway to meet the NADPH demand of the antioxidant defense system (Hayes et al. 2020). This metabolic shift results in reduced production of pyruvate, the end product of glycolysis, consequently leading to a decline in acetyl-CoA supply. As the TCA cycle is the main pathway for cellular ATP generation and acetyl-CoA serves as its key carbon substrate (He et al. 2019), insufficient acetyl-CoA supply directly impairs the efficiency of mitochondrial ATP synthesis (Obata and Fernie 2012). Similar metabolic reprogramming has been reported in *Arabidopsis thaliana* (Tang et al. 2025).

This study confirmed that Cd^2^^+^ stress significantly reduced the accumulation levels of acetyl-CoA and ATP in cotton leaves. Notably, the pYL156:*GhBCAT12* lines exhibited a more severe metabolic depletion phenotype under Cd^2^^+^ treatment, with their acetyl-CoA contents further decreased compared with the pYL156 control group. This phenomenon aligns well with the existing theoretical framework: research indicates that one of the core challenges plants face under stress conditions is energy deprivation, and when soluble carbohydrate reserves are depleted, plants mobilize alternative respiratory substrates by activating catabolic pathways (including autophagy and the degradation of lipids and proteins) to maintain basal ATP supply (Heinemann and Hildebrandt 2021; Neinast et al. 2019). In this state of energy deprivation, amino acid catabolism, particularly the degradation of BCAAs, plays a crucial compensatory role as an important alternative source of acetyl-CoA (Yao et al. 2017). In fact, in *Arabidopsis*, the complete oxidation of BCAAs efficiently generates ATP through oxidative phosphorylation (Schertl et al. 2017). Compared with other amino acids, BCAAs exhibit a particularly high ATP-producing capacity (Hildebrandt et al. 2015). This metabolic strategy of maintaining mitochondrial function by mobilizing alternative carbon sources is highly conserved across the biological world. For example, in mammalian Treg cells, when glycolysis is impaired due to inhibition of the non-oxidative pentose phosphate pathway, the cells similarly enhance amino acid catabolism and fatty acid oxidation to maintain oxidative phosphorylation levels and cellular function (Liu et al. 2022). This further confirms the universal biological significance of BCAA degradation as an energy compensation pathway.

Vacuolar sequestration is one of the core strategies for Cd^2^^+^ tolerance in plants: besides Cd^2^^+^ immobilization by the cell wall, the vacuole serves as the primary storage compartment for Cd^2^^+^ (Zhang et al. 2021). The vacuolar sequestration of Cd^2^^+^ is precisely regulated by a series of Cd^2^^+^-related transporters (Zhang et al. 2023). Currently, multiple transporter families responsible for translocating Cd^2^^+^ from the cytoplasm into the vacuole have been identified in plant root and leaf cells, including ABC transporters and heavy metal ATPases (HMAs) (Liu et al. 2024; Yu et al. 2024b). The normal function of these vacuolar Cd^2^^+^ transporters is dependent on energy supply.

Subcellular Cd^2^^+^ distribution analysis revealed that, compared with the control (pYL156), *GhBCAT12*-silenced plants exhibited significantly higher Cd^2+^ contents in the cytoplasm, whereas Cd^2^^+^ contents in the organelle fraction were significantly reduced (Fig. 5I). Considering that the vacuole is the primary organelle for heavy metal storage in mature plant cells and occupies most of the cellular volume, the decrease in Cd^2^^+^ contents in the organelle fraction likely reflects a reduction in vacuolar sequestration efficiency. In addition, Cd^2+^ contents in the shoots of silenced plants were higher than that in the control. However, the underlying causes of this increased Cd^2^^+^ accumulation in shoots remain unclear and warrant further investigation.

Based on this, we hypothesize that the acetyl-CoA depletion caused by *GhBCAT12* silencing, and the subsequent ATP supply deficiency, may directly compromise the vacuolar sequestration capacity for Cd^2^^+^. This would result in more Cd^2^^+^ remaining in the cytoplasm, thereby exacerbating Cd^2^^+^ toxicity. This explains why pYL156:*GhBCAT12* plants exhibit more severe oxidative damage and cell death. This conclusion was further supported by exogenous ATP supplementation experiments. Notably, exogenous ATP application alleviated the Cd^2^^+^-sensitive phenotype of *GhBCAT12*-silenced plants and enhanced vacuolar Cd^2^^+^ sequestration (Fig. 6). These observations provide causal evidence linking ATP depletion directly to impaired vacuolar Cd^2+^ sequestration.

### Ecological significance and application potential of *GhBCAT12* in cotton Cd^2^⁺ adaptation

Based on the above mechanistic study, *GhBCAT12* is confirmed to be a key gene in the endogenous Cd^2+^ tolerance mechanism of cotton. As a non-food crop primarily used for industrial purposes, cotton possesses inherent advantages over other crops for remediating Cd^2^^+^-contaminated soil, effectively preventing Cd^2^^+^ from entering the food chain (Wei et al. 2024). However, yield reduction caused by Cd^2^^+^ stress limits this application potential. This study identified *GhBCAT12* as a key component of the endogenous Cd^2^^+^ tolerance mechanism in cotton, a finding with significant application value. On the one hand, *GhBCAT12* could serve as a molecular marker for screening Cd^2^^+^-tolerant cotton germplasm resources. On the other hand, moderately enhancing *GhBCAT12* expression or optimizing its enzymatic activity through gene editing technologies may facilitate the development of novel cotton varieties that combine Cd^2^^+^ tolerance with low Cd^2^^+^ accumulation. It should be noted that although preliminary information regarding gene function was obtained through VIGS‑mediated silencing, this approach has inherent limitations (Zulfiqar et al. 2023). These limitations warrant further validation using stable genetic approaches. Furthermore, the conclusion regarding the substrate preference of *GhBCAT12* is based on indirect in vivo metabolic evidence, as no in vitro enzyme activity assay was performed in this study. The biochemical function of *GhBCAT12* as a BCAA aminotransferase and its substrate specificity for leucine and isoleucine therefore remain to be directly validated by enzymatic assays in future studies.

## Conclusion

**Fig. 7:**
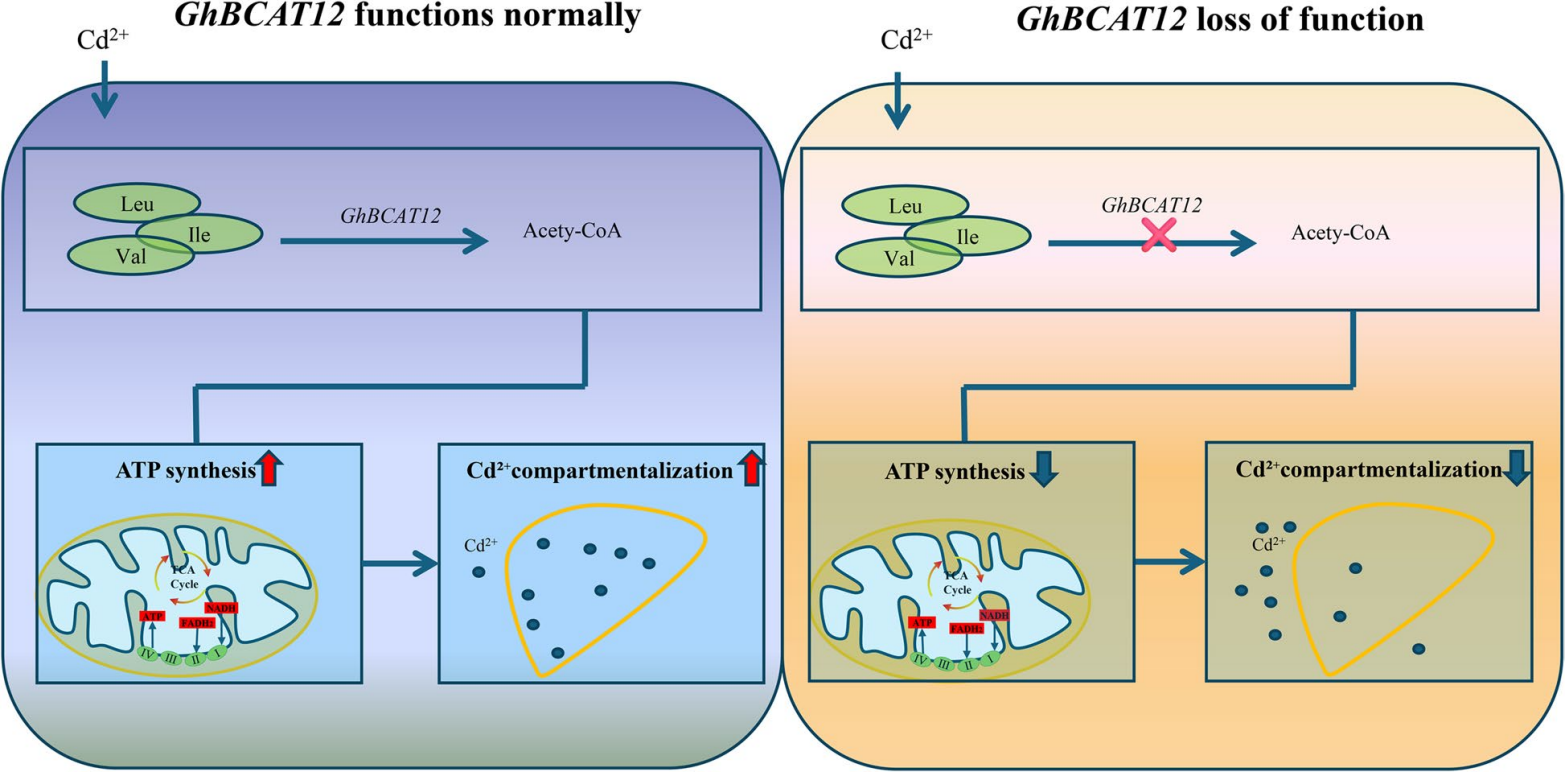
Response mechanism of *GhBCAT12* under Cd^2^^+^ stress

In this study, the *BCAT* gene family in cotton was systematically characterized, and the role of *GhBCAT12* in Cd^2^^+^ tolerance was elucidated. *GhBCAT12* was induced by Cd^2^^+^, and its silencing increased Cd^2^^+^ sensitivity, oxidative damage, and cell death. Metabolic and energy analyses revealed that *GhBCAT12* mediates BCAA degradation to supply acetyl-CoA for the TCA cycle, sustaining ATP production and vacuolar Cd^2^^+^ sequestration (Fig. 7). These findings identify *GhBCAT12* as a key regulator in cotton that links BCAA catabolism to energy metabolism and heavy metal tolerance, providing a potential target for improving crop resistance to heavy metal stress.

## Materials and methods

### *BCAT* family identification and phylogenetic analysis

Genome files for *Gossypium hirsutum* (v2.1), *Gossypium barbadense* (v1.1), *Gossypium arboreum* (v1.0), and *Gossypium raimondii* (v2.0) were downloaded from the CottonFGD database. Genome files for *Arabidopsis thaliana*, *Theobroma cacao*, *Glycine max*, *Populus trichocarpa*, and *Vitis vinifera* were downloaded from the Ensembl Plants database. Tissue expression profiles and gene expression data under various stress conditions were obtained from the Cotton Omics Database.

This study is based on transcriptomic data of the Cd^2^^+^-tolerant cotton cultivar 'Zhong H242' under Cd^2^^+^ stress from our previous study (Han et al. 2019). Through screening for differentially expressed genes and functional enrichment analysis, the *BCAT* family genes in BCAA degradation pathway were found to exhibit a significant response to Cd^2^^+^ stress. To systematically identify the members of the cotton *BCAT* gene family, the hidden Markov model (HMM) corresponding to the conserved domain PF01063 from the Pfam database was first used for preliminary screening to obtain *BCAT* candidate proteins. Subsequently, local BLAST searches were performed to further identify all potential members, and the NCBI batch Web CD-Search tool was used to validate domain integrity. Sequences lacking complete N-terminal or C-terminal conserved domains were manually excluded. Finally, 16 *BCAT* family genes were identified in *Gossypium hirsutum*. Using these 16 sequences as probes, *BCAT* homologous genes were identified in other species, namely *Arabidopsis thaliana*, *Populus trichocarpa*, *Glycine max*, *Theobroma cacao*, and *Vitis vinifera*, via local BLAST.

Sequences from the four cotton species (*Gossypium hirsutum*, *Gossypium barbadense*, *Gossypium arboreum*, and *Gossypium raimondii*), as well as from *Arabidopsis thaliana*, *Populus trichocarpa*, *Glycine max*, *Theobroma cacao*, and *Vitis vinifera*, were aligned via MEGA7 (Kumar et al. 2016), and a phylogenetic tree was constructed via the Neighbor-Joining (NJ) method.

### Chromosomal locations of *BCAT* family members

Chromosomal mapping of *BCAT* genes in the four cotton species (*Gossypium hirsutum*, *Gossypium barbadense*, *Gossypium arboreum*, and *Gossypium raimondii*) was performed and visualized via TBtools (Chen et al. 2023).

### Conserved protein motif and gene structure analysis

Conserved motifs of the genes were predicted via the MEME website, with the maximum number of motifs to find set to 10 and other parameters left at their default values. The phylogenetic relationships, conserved motifs, and gene structures were visualized via TBtools.

### *cis*-acting element analysis

Promoter DNA sequences were extracted from the CottonFGD database, spanning the 2000 bp region upstream of the translation start codon for each *GhBCAT* gene. *cis*-acting elements within the promoter regions of the *BCAT* family members in *Gossypium hirsutum* were systematically predicted via the PlantCARE online platform. The analysis focused on three functional categories of elements: those involved in regulating plant growth and development, those responsive to abiotic stresses, and those mediating hormone signal transduction. Visualization of all predicted elements was performed via TBtools (Chen et al. 2023).

### RNA extraction and expression analysis

To elucidate the temporal response and tissue-specific expression patterns of *GhBCAT* family members under Cd^2^^+^ stress, the *Gossypium hirsutum* cultivar 'Zhong H242' was selected for greenhouse cultivation. Plants were grown in a mixed substrate of nutrient soil and vermiculite under strictly controlled environmental conditions of 25 °C and a photoperiod of 16 h light/8 h dark. The expression dynamics of *GhBCAT* genes were systematically monitored by establishing four stress time points: 0 h, 6 h, 12 h, and 24 h. Specifically, cotton seedlings at the three-leaf stage were subjected to Cd^2+^ stress by applying 4 mM CdCl₂ solution, and root and leaf tissue samples were collected at the aforementioned time points. cDNA templates were obtained through RNA extraction and reverse transcription. Sixteen pairs of *GhBCAT*-specific primers were designed using the NCBI Primer-BLAST (Table S3). Subsequent gene cloning and amplification were performed using 2 × Phanta UniFi Master Mix (Dye Plus) (Vazyme, P526), and qRT-PCR analysis was conducted using the Bio-Rad 7500 Fast real-time PCR system. Relative gene expression levels were calculated using the 2^−ΔΔCT^ method (Livak and Schmittgen 2001).

### Subcellular localization of GhBCAT12

Subcellular localization of GhBCAT12 was predicted using the Target P‑2.0 online tool. For experimental validation, the coding sequence of *GhBCAT12* was cloned into a GFP expression vector digested with XbaI and KpnI. The resulting construct, along with a mitochondrial marker plasmid, was introduced into *Agrobacterium tumefaciens* strain GV3101 by electroporation. The transformed *Agrobacterium tumefaciens* were cultured at 25 °C for two days. A loop of bacterial colonies was scraped from the solid culture medium and inoculated into 10 mL of yeast extract broth (YEB) liquid medium containing kanamycin and rifampicin, followed by incubation at 170 rpm for 1 h. The bacterial cells were harvested by centrifugation at 4000 rpm for 4 min and resuspended in 10 mM MgCl₂ supplemented with 120 μM acetosyringone to an optical density at 600 nm (OD₆₀₀) of approximately 0.6. For co-localization assays, the resuspended cultures containing the mitochondrial marker and the *GhBCAT12*-GFP construct were mixed at a 1:1 ratio prior to infiltration. The mixed *Agrobacterium* suspension was infiltrated into leaves of well‑grown *Nicotiana benthamiana* plants. The infiltrated plants were incubated under low light for two days, and fluorescence signals were observed using a laser scanning confocal microscope. This experiment was performed with three independent biological replicates. In each replicate, three tobacco leaves were infiltrated. Representative images from three independent experiments are shown.

### Functional validation of *GhBCAT12* using VIGS

To validate the biological function of *GhBCAT12*, this study employed VIGS technology to achieve targeted gene silencing in cotton plants. A specific 300 bp silencing fragment for the *GhBCAT12* gene was designed using the SCN-VIGS online platform. This fragment was digested with SacI and BamHI and directionally cloned into the pYL156 vector, which had been linearized with the same restriction enzymes, to construct the recombinant silencing vector. The verified recombinant plasmid was transformed into *Agrobacterium tumefaciens* strain GV3101 competent cells.

*Agrobacterium* cultures containing pYL156 (empty vector control), pYL156:*GhBCAT12* (gene silencing), pYL156:PDS (positive control), and the helper vector pYL192 were grown to an OD_600_ of approximately 1.2. The bacterial cells were collected by centrifugation and resuspended in a buffer containing 10 mM MES, 200 μM acetosyringone, and 10 mM MgCl₂ (pH 5.8) to adjust the concentration to OD_600_ = 1.0. The suspensions were then incubated in darkness at room temperature for 4 h for induction. Subsequently, each of the three bacterial suspensions (pYL156, pYL156:*GhBCAT12*, and pYL156:PDS) was mixed with the helper vector suspension in equal volumes to prepare the inoculation solutions. The mixed bacterial solutions were respectively inoculated into the cotyledons of cotton seedlings using the injection infiltration method. After inoculation, the plants were cultivated in darkness at 25 °C for 24 h, and then transferred to a controlled-environment growth chamber with a photoperiod of 16 h light (25 °C)/8 h dark (23 °C). Successful establishment of the VIGS system was confirmed upon the appearance of the typical albino phenotype in the pYL156:PDS control group plants. Then, uniformly growing plants from the pYL156 empty vector control group and the pYL156:*GhBCAT12* gene-silenced group were selected and treated with a 4 mM Cd^2^^+^ solution for functional validation. Data are presented as mean ± SD (*n* = 3 biological replicates, with each replicate consisting of 5 plants). Statistical significance was determined by one‑way ANOVA followed by Tukey's HSD test; different letters indicate significant differences at p < 0.05.

### Physiological parameter determination and staining

To compare the differential responses of physiological and biochemical indicators in cotton seedlings under Cd^2^^+^ stress, four treatment groups were established for systematic comparative analysis in this study: empty vector control (pYL156), target gene silencing control (pYL156:*GhBCAT12*), Cd^2^^+^ stress with empty vector (Cd^2^^+^-pYL156), and Cd^2^^+^ stress with target gene silencing (Cd^2^^+^-pYL156:*GhBCAT12*). Three independent biological replicates were set for each treatment. For each sampling, 0.1 g of fresh leaves from *Gossypium hirsutum* was collected for indicator measurement. Physiological parameters were quantified using standardized assay kits according to the manufacturers' protocols: malondialdehyde (MDA) content assay kit (Solarbio, BC0025), hydrogen peroxide (H₂O₂) content assay kit (Solarbio, BC3595), superoxide dismutase activity (SOD) assay kit (Solarbio, BC0175), acetyl-CoA content assay kit (Solarbio, BC0985), ATP content assay kit (Solarbio, BC0305), and NAD(H) content assay kit (Solarbio, BC0310).

For histochemical staining, the accumulation of superoxide anions was detected using the nitroblue tetrazolium (NBT) staining method. Young leaves at consistent developmental stages, collected before and after Cd^2^^+^ treatment, were immersed in NBT staining solution (Coolaber, SL18061). The samples were vacuum-infiltrated and then stained in darkness for 2 h, followed by decolorization with 95% ethanol to observe the blue formazan precipitate. Concurrently, cell viability was assessed using the trypan blue staining method with a plant tissue staining kit (Solarbio, G4808), which specifically stains necrotic cells and reveals tissue damage areas on the leaves.

Quantitative analysis of free BCAAs was performed using high-performance liquid chromatography (HPLC). The detection service was provided by Suzhou Michy Biomedical Technology Co., Ltd. Cd^2+^ contents were determined using a quantification technique provided by Nanjing Hanguang Testing Technology Co., Ltd.

### Exogenous ATP supplementation assay

To investigate whether exogenous ATP supplementation alleviates the Cd^2^^+^-sensitive phenotype induced by *GhBCAT12* silencing, *GhBCAT12*-silenced plants (pYL156:*GhBCAT12*) were used, and two treatment groups were established. The control group consisted of silenced plants treated with 4 mM Cd^2+^, while the treatment group comprised silenced plants treated with 4 mM Cd^2+^ supplemented with exogenous ATP at various concentrations. All plants were maintained under uniform growth conditions (25 °C, with a photoperiod of 16 h light/8 h dark) after treatment. Phenotypic observations were conducted during the treatment period, and leaf samples were collected at 48 h post-treatment for subsequent analyses of physiological and biochemical parameters.

### Data analysis

All data were obtained from at least three biological replicates. Data analysis was based on a completely randomized single-factor experimental design. For comparisons between two groups, statistical significance was determined using unpaired two‑tailed Student’s t‑test. For comparisons among multiple groups, one-way analysis of variance (ANOVA) followed by Tukey's HSD test was applied, and different lowercase letters indicate significant differences at *p* < 0.05.

## Supplementary Information

Supplementary Material 1: Table S1 Renaming of *BCAT* family members in *Gossypium hirsutum, Gossypium barbadense*, *Gossypium arboreum*, and *Gossypium raimondii.* Table S2 Physicochemical properties of members of the *GhBCAT* family*.* Table S3 Primers used for qRT-PCR*.* Fig.S1 Chromosome mapping of the *BCAT* family members*.* Fig.S2 Phylogenetic analysis, conserved motifs, and gene structure of *BCATs.* Fig.S3 *cis*-acting element analysis of the promoter regions of the *GhBCAT* gene family*.* Fig.S4 TCA cycle activity assessment in *GhBCAT12*-silenced plants under Cd^2^^+^ stress*.* Fig.S5 Phenotypes of pYL156:*GhBCAT12* plants treated with various concentrations of exogenous ATP under Cd^2^^+^ stress*.* Fig.S6 Silencing efficiency of *GhBCAT12* in roots and off‑target detection of *GhBCAT4* in leaves.

## Data Availability

All data and materials are available in the paper and online supplemental files.

## Abbreviations

BCAA: Branched-chain amino acid
TCA: Tricarboxylic acid
ROS: Reactive oxygen species
BCAT: Branched-chain amino acid aminotransferase
mTP: Mitochondrial transit peptide
SP: Secretion pathway
VIGS: Virus-induced gene silencing
HMAs: Heavy metal ATPases
HMM: Hidden Markov model
NJ: Neighbor-Joining
YEB: Yeast extract broth
MDA: Malondialdehyde
H₂O₂: Hydrogen peroxide
SOD: Superoxide dismutase
NBT: Nitroblue tetrazolium
HPLC: High-performance liquid chromatography
ANOVA: Analysis of variance
