# Supplementary material for: Cadmium tolerance mediated by GhBCAT12 through branched-chain amino acid degradation via vacuolar sequestration in cotton
Source: Stress Biol. 2026 Aug 10;6(1):59. doi: 10.1007/s44154-026-00335-z (PMC13457532; doi:10.1007/s44154-026-00335-z)
Supplement: Supplementary file 1 [file 44154_2026_335_MOESM1_ESM.docx]

**Supplement Tables**

**Table S1** Renaming of *BCAT* family members in *Gossypium hirsutum,* *Gossypium barbadense*, *Gossypium arboreum*, and *Gossypium raimondii*

| **Gene ID** | **rename** | **Gene ID** | **rename** | **Gene ID** | **rename** |
| --- | --- | --- | --- | --- | --- |
| *GH_A01G1942* | *GhBCAT1* | *GB_A01G2041* | *GbBCAT1* | *Ga02G1262.1* | *GaBCAT1* |
| *GH_A01G1947* | *GhBCAT2* | *GB_A01G2046* | *GbBCAT2* | *Ga02G1266.1* | *GaBCAT2* |
| *GH_A02G0871* | *GhBCAT3* | *GB_A02G0865* | *GbBCAT3* | *Ga03G0936.1* | *GaBCAT3* |
| *GH_A05G2136* | *GhBCAT4* | *GB_A05G2168* | *GbBCAT4* | *Ga05G2221.1* | *GaBCAT4* |
| *GH_A05G2650* | *GhBCAT5* | *GB_A05G2687* | *GbBCAT5* | *Ga05G2805.1* | *GaBCAT5* |
| *GH_A06G0273* | *GhBCAT6* | *GB_A06G0278* | *GbBCAT6* | *Ga06G0285.1* | *GaBCAT6* |
| *GH_A11G2243* | *GhBCAT7* | *GB_A11G2290* | *GbBCAT7* | *Ga11G1770.1* | *GaBCAT7* |
| *GH_A13G1274* | *GhBCAT8* | *GB_D01G2135* | *GbBCAT8* | *Ga13G1359.1* | *GaBCAT8* |
| *GH_D01G2040* | *GhBCAT9* | *GB_D01G2140* | *GbBCAT9* | *Gorai.002G212000.1* | *GrBCAT1* |
| *GH_D01G2045* | *GhBCAT10* | *GB_D02G0937* | *GbBCAT10* | *Gorai.002G212600.1* | *GrBCAT2* |
| *GH_D02G0891* | *GhBCAT11* | *GB_D05G2189* | *GbBCAT11* | *Gorai.005G098300.1* | *GrBCAT3* |
| *GH_D05G2168* | *GhBCAT12* | *GB_D05G2687* | *GbBCAT12* | *Gorai.007G227600.1* | *GrBCAT4* |
| *GH_D05G2671* | *GhBCAT13* | *GB_D06G0270* | *GbBCAT13* | *Gorai.009G223000.1* | *GrBCAT5* |
| *GH_D06G0256* | *GhBCAT14* | *GB_D11G2242* | *GbBCAT14* | *Gorai.009G277800.1* | *GrBCAT6* |
| *GH_D11G2204* | *GhBCAT15* | *GB_D13G1289* | *GbBCAT15* | *Gorai.010G028900.1* | *GrBCAT7* |
| *GH_D13G1207* | *GhBCAT16* | */* | */* | *Gorai.013G123700.1* | *GrBCAT8* |

**Table S2** Physicochemical properties of members of the *GhBCAT* family

| Gene ID | Rename | Protein Length  (aa) | Molecular Weight  (Da) | Isoelectric Point  （PI） |
| --- | --- | --- | --- | --- |
| *GH_A01G1942.1* | *GhBCAT1* | 355 | 39191.1 | 6.0 |
| *GH_A01G1947.1* | *GhBCAT2* | 387 | 43060.7 | 8.0 |
| *GH_A02G0872.1* | *GhBCAT3* | 412 | 45456.3 | 8.7 |
| *GH_A05G2136.1* | *GhBCAT4* | 385 | 42465.2 | 8.1 |
| *GH_A05G2650.1* | *GhBCAT5* | 390 | 42792.7 | 7.5 |
| *GH_A06G0273.1* | *GhBCAT6* | 384 | 42239.4 | 6.7 |
| *GH_A11G2243.1* | *GhBCAT7* | 425 | 46452.2 | 6.5 |
| *GH_A13G1274.1* | *GhBCAT8* | 242 | 26812.7 | 8.7 |
| *GH_D01G2040.1* | *GhBCAT9* | 305 | 33258.5 | 8.4 |
| *GH_D01G2045.1* | *GhBCAT10* | 387 | 42986.6 | 8.0 |
| *GH_D02G0891.1* | *GhBCAT11* | 333 | 36444.7 | 5.6 |
| *GH_D05G2168.1* | *GhBCAT12* | 385 | 42574.3 | 8.1 |
| *GH_D05G2671.1* | *GhBCAT13* | 381 | 41826.6 | 7.5 |
| *GH_D06G0256.1* | *GhBCAT14* | 384 | 42040.1 | 6.6 |
| *GH_D11G2204.1* | *GhBCAT15* | 425 | 46365.2 | 8.1 |
| *GH_D13G1207.1* | *GhBCAT16* | 560 | 63085.3 | 6.7 |

**Table S3** Primers used for qRT-PCR

| **Serial number** | **name of Primers** | **Primers for qRT-PCR(5'-3')** |
| --- | --- | --- |
| 1 | *GhBCAT1*-F | CAGTGGGCAATGGTCTCAAG |
|  | *GhBCAT1*-R | GGGCGAGTAATTCGTGACAG |
| 2 | *GhBCAT2*-F | CCAGGTTGAGGAACGAGCTA |
|  | *GhBCAT2*-R | AGCTTGTCCTCGATCAGACC |
| 3 | *GhBCAT3*-F | AAAGAAGATGGGAGGCTGCT |
|  | *GhBCAT3*-R | CATGTATTCAGGCGCTGGAC |
| 4 | *GhBCAT4*-F | ATGAGTACGCGGATGTGGAT |
|  | *GhBCAT4*-R | CCTGCGGAAGGGCTTATTTC |
| 5 | *GhBCAT5*-F | TCAACTCCGGCAACAAATGG |
|  | *GhBCAT5*-R | GGCACACCAATCTACCCTCTCT |
| 6 | *GhBCAT6*-F | ACGAGTACTGTATCGGCGAA |
|  | *GhBCAT6*-R | GCCTGTGCCTTCATAATCCC |
| 7 | *GhBCAT7*-F | TGCTTGATGCTGACGAAGTG |
|  | *GhBCAT7*-R | AGACCCATCTGGAGTCTGGT |
| 8 | *GhBCAT8*-F | GCTTCCCGAATGGAAACTCC |
|  | *GhBCAT8*-R | TGCTCGAGTAATCCCAGGAA |
| 9 | *GhBCAT9*-F | CCAGGTTGAGGAACGAGCTA |
|  | *GhBCAT9*-R | TGTCCTCGATCACACCAGTT |
| 10 | *GhBCAT10*-F | GCACAGGAACAGCTATGGTG |
|  | *GhBCAT10*-R | AGCTTGTCCTCGATCAGACC |
| 11 | *GhBCAT11*-F | TATTGAGATTGCGCGTGGTC |
|  | *GhBCAT11*-R | TACAAGAGTGGAGCCCAGTG |
| 12 | *GhBCAT12*-F | ATGTGCATGCCTTCACCTTC |
|  | *GhBCAT12*-R | AGGACCTGTTCCCACAAGAA |
| 13 | *GhBCAT13*-F | GTTGGAGTTGCTCTGGTTGG |
|  | *GhBCAT13*-R | ACCAGTTTGAAGTCCCACGA |
| 14 | *GhBCAT14*-F | ATTGCGAATGAGGGTTGGTG |
|  | *GhBCAT14*-R | TCCAAGGGTTGCACGATGTA |
| 15 | *GhBCAT15*-F | TCGTGGTGAAGGGTAATGTCA |
|  | *GhBCAT15*-R | AACCATCTACGCCATACGGT |
| 16 | *GhBCAT16*-F | CAGTTATCGATGCCGCAGAC |
|  | *GhBCAT16*-R | CTGCCTCACGTTGTGCTTTA |
| 17 | Actin-F | ATCCTCCGTCTTGACCTTG |
|  | Actin-R | TGTCCGTCAGGCAACTCAT |
| 18 | *GhCS6*-F | CGCTATCCAGGCTAAGGTCA |
|  | *GhCS6*-R | GTTTCCCAAAGCAACCCTGT |
| 19 | *GhIDH1*-F | AGCTGAATATGCCGTGTTCG |
|  | *GhIDH1*-R | CTTCCTGGGTAGTGCTTTGC |

**Supplement Figures**

**Fig.S1** Chromosome mapping of the *BCAT* family members. **A** Chromosomal localization of *BCAT* genes in *Gossypium hirsutum.* **B** Chromosomal localization of *BCAT* genes in *Gossypium barbadense.* **C** Chromosomal localization of *BCAT* genes in *Gossypium arboreum.* **D** Chromosomal localization of *BCAT* genes in *Gossypium raimondii.*

**Fig.S2** Phylogenetic analysis, conserved motifs, and gene structure of *BCATs*. **A** Phylogenetic tree of *BCAT* genes. **B** Conserved motifs of *BCAT* genes. **C** Exon-intron structure of *BCAT* genes.

**Fig. S3** *cis*-acting element analysis of the promoter regions of the *GhBCAT* gene family. **A** Phylogenetic tree of the *GhBCAT* gene family. **B** *cis*-acting elements in the promoters of the *GhBCAT* gene family.

**Fig. S4** TCA cycle activity assessment in *GhBCAT12*-silenced plants under Cd²⁺ stress. **A** Relative transcript levels of *GhCS6*. **B** Relative transcript levels of *GhIDH1*. **C** NADH/NAD⁺ ratio. Data are presented as mean ± SD (n = 3 biological replicates). Statistical significance was determined by Student’s t‑test (**p < 0.01, ns indicates no significant difference).

**Fig. S5** Phenotypes of pYL156:*GhBCAT12* plants treated with various concentrations of exogenous ATP under Cd²⁺ stress. Representative images from three independent biological replicates are shown.

**Fig. S6** Silencing efficiency of *GhBCAT12* in roots and off‑target detection of *GhBCAT4* in leaves. **A** relative transcript levels of *GhBCAT12* in roots. **B** *GhBCAT4* in leaves of pYL156 and pYL156:*GhBCAT12*‑silenced plants. Data are presented as mean ± SD (n = 3 biological replicates). Statistical significance was determined by Student’s t‑test (ns indicates no significant difference).
